# The Perceptions of New Zealand Lawyers and Social Workers About Children Being Adopted by Gay Couples and Lesbian Couples

**DOI:** 10.3389/fpsyg.2020.520703

**Published:** 2020-09-25

**Authors:** Rhoda Scherman, Gabriela Misca, Tony Xing Tan

**Affiliations:** ^1^Department of Psychology, Auckland University of Technology, Auckland, New Zealand; ^2^School of Psychology, University of Worcester, Worcester, United Kingdom; ^3^Department of Educational and Psychological Studies, University of South Florida, Tampa, FL, United States; ^4^Department of English, School of Foreign Studies, Xi’an Jiaotong University, Xi’an, China

**Keywords:** same-gender parenting, lesbian and gay parents, adoption by same-gender couples, same-sex adoption, lawyers, social workers, adoption professionals’ attitudes, New Zealand

## Abstract

Global trends increasingly appear to be legitimizing same-gender relationships, yet international research shows that despite statutory rights to marry—and by extension, adopt children—same-gender couples continue to experience difficulties when trying to adopt. Primary among these barriers are the persistent heteronormative beliefs, which strongly underpin the unfounded myths about parenting abilities of same-gender couples. Such biased beliefs are perpetuated by some adoption professionals who oppose placing children with lesbian or gay couples. In 2013, New Zealand passed the Marriage Equality Act, making it possible for same-gender couples to legally marry—and by extension, adopt. This provided an opportunity to investigate the perceptions of New Zealand professionals about children being placed with same-gender couples, in a country often perceived to be more tolerant of LGBT people. New Zealand social workers and lawyers (an under-studied group)—the professions most likely involved in adoption—were recruited via professional bodies. Because studying perceptions and beliefs on socially sensitive topics are highly susceptible to social desirability, we designed an instrument utilizing multiple methods to assess and corroborate participants’ views about placing children for adoption with couples of the same gender. Administered online and anonymously, the survey included demographic questions, evaluation of negative-meaning and positive-meaning statements, and used a scenario describing a prospective adoptive couple whose gender was ambiguous, in the context of adopting children of varying needs. Overall, the study found that while New Zealand lawyers and social workers (*N* = 314) had generally favorable views of gay and lesbian adoption, they still reported a preference to see children adopted by heterosexual couples over same-gender couples, within which lesbian and gay couples were preferred equally. Moreover, being religious and politically conservative were characteristics associated with more negative views toward placing children with same-gender couples. We conclude that, despite winning the rights to marry (and adopt as couples), such legislative wins might be merely the first hurdle to be overcome; normalizing same-gender parenting is what needs to happen next. Our study adds to the research focused on adoption professionals in various countries, with the ultimate aim to inform practices and policies supportive of families headed by same-gender couples and formed through adoption.

## Introduction

On 17th April, 2013, New Zealand became the 13th country to give same-gender couples the right to marry (Chapman, 2013). Commensurate and implicit in this law change, lesbian and gay couples also became eligible to adopt *as couples*, whereas prior to the law change, only married couples (i.e., heterosexual couples) although in some cases, single persons could adopt children in New Zealand (Gibbs and Scherman, 2013). These socio-political changes mirror similar trends that are increasingly legitimizing same-gender relationships in nearly 30 countries (Masci et al., 2019).

In this climate, a question arose: is the adoptive parenting by same-gender couples also being legitimized, given that the right to adopt as a couple is so often predicated on the requirement of being lawfully married? A look through the international research literature shows that despite numerous countries awarding statutory rights to marry—and by extension, adopt—lesbian and gay couples continue to report facing ongoing obstacles when trying to adopt, perpetrated mainly by individual adoption worker and agency biases (e.g., Brodzinsky, 2003; Ryan et al., 2004; Matthews and Cramer, 2006; Sullivan and Harrington, 2009; Kinkler and Goldberg, 2011; Messina and D’Amore, 2018).

Can we expect the same trend in New Zealand? Is the passage of laws allowing same-sex marriage seemingly reflective of positive perceptions of same-gender parenting? These are the overarching questions that incited the current study. While legislation might make it possible for lesbian and gay couples to adopt, it remains unclear how professionals feel about children being placed with lesbian couples or gay couples. Therefore, the present study set out to explore New Zealand professionals’ perceptions of lesbian and gay couples adopting children. The paper first summarizes the prevailing myths—and refuting evidence—about same-gender parenting and adoption, in order to better understand the biases against adoptions by same-gender couples. This is followed by a review of the international research identifying the primary barriers experienced by prospective same-gender adopters. New Zealand’s unique context is then considered, before describing the study.

### Myths and Stereotypes Surrounding Same-Gender Parenting

Despite some progressive social changes that reflect more accepting attitudes toward gay and lesbian couples, doubts remain as to their ability to successfully parent or adopt (Montero, 2014). In short, same-gender parenting remains a contentious and polarizing issue, fuelled in large part by widespread hetero-normative assumptions that the “*married, two-parent, heterosexual couple [is] the norm against which all other kinds of couples are measured, evaluated, and judged”* (Lubbe, 2008, p. 326). These heterosexist beliefs lead to (mis)perceptions that families headed by same-gender couples are different—if not dangerous. At the same time, a type of *homo-normative* representation is also reinforced, whereby the acceptable homosexual is one that most resembles the heterosexual (Appell, 2008; Riggs, 2012).

These residual homophobic attitudes and sexist beliefs are strongly underpinned by religious fundamentalism, Christian orthodoxy and political conservatism (Rowatt et al., 2006; Jonathan, 2008). Furthermore, religiosity (defined here as *the quality of being religious;* Dictionary.com, 2020) has a clear representation of what *family* should look like: a married man and women, with biologically related children—which fundamentally contradicts the model of families headed by same-gender couples (Brown et al., 2009; Rye and Meaney, 2010; Sohr-Preston et al., 2017). This brings sexual orientation to the forefront of the debate about what makes an appropriate family and suitable parents for children, and perpetuates long-standing myths and misperceptions, including that:

1. Children need both male and female role models, which parents of the same-gender do not provide.
2. Children raised by same-gender parents will be maladjusted and suffer stigma, social harm, and bullying.
3. Children of same-gender parents will become gay or suffer gender identity confusion.
4. Gay men (in particular) and lesbian women are more likely to sexually abuse their children.

### Researchers Refute Myths and Stereotypes

In response to the myth that only when being raised by a mother and a father can children grow to be well-adjusted adults, research from the United Kingdom and United States reported that families headed by same-gender couples regularly engage with, and gain support from, large networks and communities of like-minded people—of both genders (Golombok et al., 2003; Erich et al., 2005; McCann and Delmonte, 2005; Gianino, 2008; Farr et al., 2010; Kinkler and Goldberg, 2011; Leddy et al., 2012). Moreover, children whose lesbian mothers or gay fathers were originally in heterosexual relationships, will also have both mothers and fathers in their lives in the same way—and with the same varying degree of contact—as children of heterosexual couples who divorce and remarry. According to Gates (2015), any disadvantage experienced by the children in families headed by same-gender couples can be explained by the instability experienced in the divorce that proceeded the same-gender union, rather than the sexual orientation of the parents.

Moreover, some authors have reported that the psychological adjustment of children with same-gender parents is not merely on par with children raised by heterosexual parents, but that children raised by lesbian and gay parents had better psychological adjustment (Biblarz and Stacey, 2010; Fedewa et al., 2015). Adding further strength to the argument that families headed by same-gender couples offer something positive for children, Appell (2008) considered the normative and non-normative features of families headed by same-gender couples, and the implications of these types of families within the context of adoption. She suggested that same-gender couples are becoming more heteronormative, in that they are opting for monogamy and marriage, and in that context, forming nuclear two-parent families. On the surface, this might seem like a good move; however, Appel argues that this style of family runs the risk of becoming too much like the less-preferred “closed” adoptive family model. Whereas, when the families of same-gender parents are truer to their natural social kinship model, owing to the fact that many same-gender couples require others outside of the two-parent relationship to create their families, this style of family mirrors that of the preferable “open adoption” model.

*To the extent that homosexual families are normative in their nuclear structure, they are in danger of falling into the trap of the closed adoption model. On the other hand, these lesbian and gay families who are not wedded to the nuclear structure are finding themselves and their children in larger genetic and social kinship networks… Adoption with contact is a model of community or shared parenting that may have lessons for these same-sex parent families. It undermines the heteronormative model of two-parent, exclusive parenting by recognizing the multiple people who have parental or parent-like relationships with children.* (Appell, 2008, pp. 309–310).

Empirical studies have drawn similar conclusions about the openness of sexual minority adoptive parents, when compared to heterosexual adoptive parents. In both domestic (Brodzinsky and Goldberg, 2016) and international (Brodzinsky and Goldberg, 2017) adoptions, sexual minority adopters were reported to have more post-placement contact with birth families, likely due to having “a more expansive notion of family” (Brodzinsky and Goldberg, 2017, p. 122) and greater emphasis on social versus biological kinship relationships. It has also been reported that some birth parents intentionally select lesbian or gay prospective adopters due to the belief that sexual minority parents embody diversity and would be more tolerant (Farr et al., 2018b).

Opponents to same-gender parenting have also argued that children raised by lesbian and gay parents will suffer the risk of social harm (Black, 2005), due to being stigmatized, harassed, or bullied by peers. While children raised by same-gender parents may experience some stigma or bullying from their peers (Crouch et al., 2016), this has been found to occur no more frequently than it does to children from heterosexual families (Vanfraussen et al., 2003; Gartrell et al., 2005). Some authors support the notion that the stresses caused by stigmatization/bullying can result in positive learning experiences for the children, enhancing their resilience and resulting in personal growth (Bos et al., 2008; Telingator, 2013; Titlestad and Pooley, 2013). Other researchers have determined that children’s well-being is more affected by *family processes* (e.g., quality of parenting) than *family structure* (e.g., number or sexual orientation of parents) (Short et al., 2007; Golombok and Tasker, 2015).

In the matter of children’s gender development, and fears that growing up with lesbian or gay parents will result in gender identity confusion, studies repeatedly refute this myth (e.g., Carone et al., 2020), finding instead that a child’s own gender, as opposed to parental sexual orientation, is a stronger influence on whether or not children engage in gender-conforming behaviors (Farr et al., 2018a). On the other hand, Gartrell et al. (2019), reporting on findings from their US longitudinal study spanning more than 20 years, did find a greater likelihood of same-sex attraction and sexual minority identity in the offspring of lesbian parents, suggesting that being raised by same-gender parents can lead to more diverse sexual expression. Importantly, it has also been suggested by other researchers that if the offspring of same-gender parents do turn out to be homosexual, the likelihood is extremely high that they will grow up in more accepting environments, than did many lesbian and gay individuals who grew up in heterosexual homes (Carastathis et al., 2017; VanderWaal et al., 2017). Regardless of the eventual sexual identity of children raised by lesbian and gay parents, the offspring are being raised in accepting environments that promote more tolerance of diversity, which many of the children/young adults themselves believe to be a beneficial by-product of their unique family life (Welsh, 2011).

Finally, one of the earliest and more denigrating myths about same-gender parenting is the notion that children raised by gay or lesbian parents are more likely to be sexually abused, which appears to stem from the belief that homosexuals are sexually deviant people (Hicks, 2006). A review of the scientific research shows no such support for these claims (Ryan and Cash, 2003; Herek, 2006; Tasker and Bellamy, 2019). Pedophilia, which is the sexual attraction of an adult to a child, is completely unrelated to the adult’s sexual orientation (Mallon, 2000). In fact, Jenny et al. (1994) reported that children are “over 100 times” (p. 44) more likely to be molested by a relative’s heterosexual partner than by an identifiably gay person.

### Barriers to Same-Gender Parenting Still Remain: A Review of Literature

The evidence in support of same-gender parenting is persuasive. With so much empirical evidence discounting the myths, coupled with eroding legal/statutory barriers, it is surprising to find that lesbian and gay couples still battle to be considered as adoptive parents. Nonetheless, research demonstrates that negative attitudes and discriminatory treatment by *adoption professionals* continues to be another significant barrier to adoption and fostering for sexual minority individuals and couples (e.g., Brooks and Goldberg, 2001; Brodzinsky et al., 2002; Brodzinsky, 2003; Ryan et al., 2004; Matthews and Cramer, 2006; Mallon, 2007; Ryan and Whitlock, 2008; Anderssen and Hellesund, 2009; Sullivan and Harrington, 2009; Kinkler and Goldberg, 2011; Goldberg et al., 2012).

Within this body of literature, reports of outright homophobia and/or deliberate discriminatory stances are rare. Instead, most of the studies reported some degree of acceptance of sexual minorities and a willingness (in principle, at least) to consider applications to adopt (or foster) by lesbian and gay individuals/couples. On the other hand, “… *there appears to be a level of subjectivity inherent in the approval process that is strongly suggestive of bias*” (Sullivan and Harrington, 2009, p. 243). Moreover, it has been reported that many agencies lack policies or guidelines for same-gender adoptions, resulting in placement decisions being made at the discretion of individual social workers, who may allow personal biases to unfairly influence the adoption process (Kenyon et al., 2003; Ryan et al., 2004). Consequently, in their bid to become adoptive parents, same-gender applicants face ongoing challenges related to religiosity, political ideology, hetero-normative biases, and differential treatment of sexual minorities by adoption workers. These barriers are briefly considered below.

#### Religiosity as a Barrier to Adoption by Same-Gender Couples

One of the strongest predictors of negativity toward same-gender adoption is religiosity (e.g., Ryan, 2000; Brodzinsky, 2003; Mallinger, 2010; McCutcheon and Morrison, 2014; Jäckle and Wenzelburger, 2015; Kimberly and Moore, 2015; Sohr-Preston et al., 2017). In this body of research, religiosity was sometimes measured in terms of the religious affiliation of the adoption agencies (e.g., Brodzinsky, 2003; Kimberly and Moore, 2015). Brodzinsky et al. (2002), for example, found that 100% of the Christian fundamentalist agencies and most Catholic-based agencies refused to work with same-gender applicants. In a subsequent study, Brodzinsky (2003) reported again that all of Christian fundamentalist and Baptist agencies (and a majority of Mormon, Catholic, and Methodist) refused to work with homosexual adopters. On the other hand, he found that Jewish-affiliated agencies and most Lutheran organizations were willing to place children with same-gender couples. More often, it has been the *religiousness* of individual staff that have been found to correlate with, or influence, placement decisions (Jäckle and Wenzelburger, 2015). For instance, Mallinger (2010) found that in a group of social workers in the United States, religious fundamentalism influenced individual attitudes toward lesbian and gay adopters, and reduced the likelihood of children being placed with same-gender prospective adopters (Mallinger, 2010).

#### The Relationship Between Political Ideology and Biases Against Adoptions by Same-Gender Couples

As noted earlier in the paper, often accompanying the Christian fundamentalist beliefs that underpin much of the homophobia experienced by prospective adopters, is a conservative or right-wing political ideology. It is a finding often seen in studies about attitudes toward homosexuality generally (e.g., Brown and Henriquez, 2008; Jäckle and Wenzelburger, 2015; Prusaczyk and Hodson, 2020), and as a barrier to the willingness of adoption professionals to work with sexual minority parents (e.g., Hall, 2010; Molina and Alarcón, 2015). For example, in their study of adoption agency directors in the United States, Kimberly and Moore (2015) reported that those who self-identified as republicans had more conflicted feelings about same-gender couples than those labeled as independent or democratic. Similarly, Jayaratne et al. (2008) from the United States, found that liberalism/conservatism was a significant predictor of whether or not the child welfare workers in that study would place children with lesbian and gay parents.

On the other hand, low religiosity and liberal political ideology, were found to accompany positive attitudes toward same-gender adoption in research from Portugal (Costa et al., 2014), and Spain (Molina and Alarcón, 2015). Finally, based on data from 28 European countries, Takács et al. (2016) also explored (among other variables and indices) the influence of political ideology and religiosity, reporting that the “*attitudes toward same-sex adoption were relatively positive among those … not bound to religious communities, … and who had a moderate left position on the right–left scale of political orientation*.” (2016, p. 1796). These studies suggest that while political conservatism may be another barrier to adoption for same-gender couples, liberal ideologies on the part of adoption professionals, may assist sexual minorities in their bid to become adoptive parents.

#### Hetero-Normative Biases Against Same-Gender Couples

Additional barriers have been reported by researchers, many of which involve what McCutcheon and Morrison (2014) refer to as *homonegativity*. This seems to reflect attitudinal alignment with stereotyped notions of lesbian and gay couples, on the part of social workers and other adoption professionals. Men, in particular, have been reported to hold the strongest homophobic or anti-gay attitudes (e.g., Ryan, 2000; Brodzinsky et al., 2002; Arnold et al., 2004; Costa et al., 2014; Kemper and Reynaga, 2015; Mirabito, 2014); however, the role of gender has not been as consistently considered across the research literature.

Social workers’ negative attitudes appear to result in those *hetero-normative* biases described earlier, which preference straight couples and result in differential treatment of gay and lesbian adopters. Also described as *institutional discrimination* (Goldberg et al., 2013) and *professional homophobia* (Ryan et al., 2004), these placement biases are often subtle but sometimes manifest as more overt forms of discrimination by adoption agency staff (Kinkler and Goldberg, 2011). In their Canadian study of social workers’ perceptions of biases at play when making placement decisions, Sullivan and Harrington (2009) illustrate this issue—as well as a type of duplicity taking place in the approval process. After initially reporting that same-gender couples were routinely being “approved,” the social worker participants in the study emphasized that “*approval does not guarantee a placement*” (Sullivan and Harrington, 2009, p. 243). The research respondents explained that while the lesbian and gay couples were regularly approved, their home study reports were being *“…written in such ways that nobody will ever accept them as adoptive families because there are enough issues identified in the study that people will not go forward and place children with them”* (p. 241). As they tried to make sense of the duplicity occurring in the approval processes, Sullivan and Harrington (2009) argued against the idea that social workers are biased in general. The authors posited instead that the social workers are “*affected by stigma by association*” (p. 242), wherein social workers may be displaying a type of “*vicarious stigma*” (p. 244), as they act in the interests of biases they know or expect to exist. Whether occurring due to first-hand or vicarious biases, the study clearly showed that same-gender applicants are routinely being passed over for the more preferable heterosexual couples.

#### Differential Treatment of Same-Gender Applicants

Other studies show that the duplicity, bias and discrimination described by Sullivan and Harrington (2009) and others, is a source of considerable stress. Prospective adopters feel over-scrutinized (Brooks and Goldberg, 2001), and left with significant feelings of self-doubt (Messina and D’Amore, 2018). Ross et al. (2008), for example, in their qualitative study of the mental health outcomes of lesbian adoptive mothers in Canada, found that one of the most significant influences on the women’s sense of wellbeing was “*subtle, insidious homophobia and/or heterosexism*” (p. 260). The authors went on to report that as a group, their participants felt they were regularly the last choice, after all heterosexual couples were considered. This type of differential treatment of same-gender couples by North American agencies was also reported by Kenyon et al. (2003) who found that if agencies did select homosexual parents, they were often only offered children with special needs. Pressure to take special needs children was also reported by Brodzinsky et al. (2002), Matthews and Cramer (2006), Goldberg et al. (2007), and Averett et al. (2009), whose American participants reported feeling that their “*social workers persisted in ‘trying to give us the most damaged kids they know no one will take*”’ (p. 53).

When considered separately, several authors suggest that prospective gay adopters are even more likely than lesbian women to experience resistance from adoption professionals. In his qualitative interviews with gay adoptive couples in the United States, Gianino (2008) described countless examples of how the men needed to negotiate their own type of duplicity that involved non-disclosure of their sexual orientation. On the other hand, to opt for openness and transparency, the gay couples risked anti-male gender biases from adoption professionals based on the belief that “*children need a mother*” (p. 216), as well as from some birth parents who rejected the idea of placing their children with a gay couple (Gianino, 2008).

Yet, while such omissions about being lesbian or gay may appear to some prospective adopters to increase their chances of successfully adopting, it will more than likely mean that only one parent will be recognized as the “legal” parent. Recall from the opening section of the paper that joint adoption by same-gender couples is almost always predicated on being legally wed. As such, in some countries, failing to disclose one’s relationship status (and therefore, one’s sexual orientation) means that only one member of the couple will be able to legally adopt. This leaves the other person as a silent, unacknowledged parent with potentially no legal standing (Blanks et al., 2004; Appell, 2008; Perrin et al., 2013).

### The Legal Standing of Gay and Lesbian Adoptive Parents

Enter the lawyers, a whole new set of professionals that prospective adopters may need to work with in their bid to become/remain parents. The lawyers’ attitudes about seeing children placed with lesbian and gay couples is seemingly non-existent in the research literature. The lack of attitudinal research on lawyers, in regards to same-gender parenting, comes in stark contrast to what is otherwise an abundance of law literature about sexual minorities. This body of work has emphasized that within the criminal justice systems, lesbian women and gay men have long been the objects of negative stereotypes, prejudice, discrimination and even violence on the basis of their sexual orientation (Williams, 2015; Knight and Wilson, 2016). We also found no shortage of scholarship on the subject of same-gender marriage, the rights of same-gender couples, and the legal parentage of their children—which was, it is important to note, all from the United States. Within that body of literature, the “best interests of the child” was a dominant theme. Another repeating theme was the vulnerability of the children when one parent has no legal standing, especially if the same-gender union ends (e.g., Joslin, 2005; Graham, 2008; Barfield, 2014; Acosta, 2017; Mason, 2018). Without a legally protected parental relationship, children can miss out on inheritance rights, retirement benefits, and even health insurance, and they can lose access to the non-legal parent in the event that the parental relationship ends (Goldberg and Kuvalanka, 2012). The non-legal parent also runs the risk of not being able to travel with the child (Perrin et al., 2013), make medical decisions for an injured child, or worse, may not be able to maintain a relationship with the child in the event of divorce or death of the legal parent (Joslin, 2005).

#### The Relationship Between Marriage Rights and Parental Rights

After our review of the law literature, one question emerged. With the legal literature so focussed on the many risks associated with same-gender couples raising children when one parent is not legally recognized, would any of these statutory issues continue to exist in the aftermath of *Obergefell v. Hodges—*the 2015 Supreme Court ruling allowing for the nation-wide legalization of same-gender marriage in the United States. Surprisingly, the short answer seems to be a resounding yes. As Esser explains:

*“Obergefell only addressed marriage rights—the relationship between the adults in a family. It did not specifically address the legal relationship of each of the parents to the children the family is raising”* (2016, p. 1).

It seems that children remain at risk when both parents do not have legally sanctioned relationships with their children (e.g., Zarembka, 2015; Esser, 2016; Harris, 2017; Vaughn, 2017), and marriage equality laws do not address this parent/child relationship. Therein lies the rub: even though adoption by most same-gender couples cannot exist without first achieving statutory rights to marry, marriage equality laws do not necessarily concern themselves with subsequent parent-child relationships. Since marriage equality laws are about the relationships of adults, and not about adult relationships to children/offspring or parenting, it may be that the legalization of same-gender marriage is not all that is needed in order to shift attitudes about same-gender parenting/adoption.

### Summary of the International Literature and Rationale for New Zealand Study

In summary, there is a robust (yet predominately North American) body of social science research that almost uniformly finds same-gender couples to be capable and competent parents, whose children are not disadvantaged from being raised by parents in same-gender relationships (e.g., Crowl et al., 2008; Fedewa et al., 2015; Patterson, 2017). The international research also shows that despite hard-won statutory rights to marry—and by extension, adopt—lesbian and gay couples continue to encounter barriers when trying to adopt, perpetrated mainly by the biased attitudes of agencies and individual adoption workers (Ryan et al., 2004; Brodzinsky, 2003; Matthews and Cramer, 2006; Sullivan and Harrington, 2009). Concerns raised in the above literature about prospective adopters not disclosing their sexual orientation led us to the law literature. Despite a wealth of articles focussed on the statutory rights and challenges facing sexual minorities and same-gender couples wishing to adopt, the attitudes of lawyers, themselves, toward same-gender parenting/adoption remains uncertain, and empirically unexplored. Our interpretation of the law literature—as it informs the subject of adoptions by same-gender couples—is that a country’s marriage-equality laws may be insufficient on their own to enable those couples to adopt. Hence, it may be that for lesbian and gay couples, winning the right to legally marry is merely the first hurdle; changing the attitudes of individual adoption workers—and lawyers—might be the next hurdle to overcome in their quest to become adoptive parents.

With the relatively recent passage of law in New Zealand, permitting lesbian and gay couples to marry—and adopt—we saw an opportunity to explore these issues outside of the dominant North American and European contexts, in a country perceived to be inclusive and progressive in its treatment of the LGBT community. Before introducing the current study, below we briefly describe New Zealand in terms of its attitudes toward sexual minorities, and describe some of the country’s social welfare and adoption systems that also set it apart from the North American contexts that currently dominate the empirical literature.

### The New Zealand Context

New Zealand is a relatively small country in the South Pacific, with a population of almost five million people (Statistics New Zealand, 2020). The country has a White European majority population as a result of colonization, and an Indigenous minority culture: the Māori. In terms of empirical research on the country’s attitudes toward homosexuality, two studies (Kelley, 2001; Smith, 2011) found New Zealand to be in the middle range of scores, relative to the other 28 and 41 countries (respectively). Importantly, both studies reported that most of the countries (New Zealand included) showed bimodal distributions in their attitudes; if the majority of people in a country either approved or disapproved of homosexuality, the second largest group often took the opposite stance. In this way, it is difficult to say what the majority of New Zealanders think about homosexuality.

In terms of prevalence of sexual minorities, Greaves et al. (2017) reported that out of a large national sample of more than 18,000 New Zealanders, 2.6% described their sexual orientation as lesbian/gay, with another 1.8% bisexual. However, the researchers used a novel approach to gather this data, offering the question of sexual orientation as an open-ended item. In so doing, the analysis began with an initial 49 different codes for how people described their sexual orientation, some of which could not be classed within the binary structure of heterosexual versus homosexual. The authors concluded that the diverse and nuanced ways that New Zealanders described their sexual orientation may reflect societal changes in how people see sexual orientation.

Regarding the status of sexual minorities in New Zealand more generally, there is evidence to suggest that New Zealand may be rather liberal when it comes to the LGBT communities. New Zealand was the first country in the world to see a transgender woman elected to the office of mayor, and shortly thereafter, she become the first openly transgender member of Parliament—both world firsts for New Zealand (Herkt, 2018; New Zealand Parliament, 2020). In fact, New Zealand has had openly gay and lesbian members of Parliament since 1993, and even the New Zealand Police and the Royal New Zealand Navy are said to have long had “gay-friendly” policies. Several online sites rank New Zealand quite high in terms of being “gay-friendly,” further illustrating how the country is perceived by LGBT people in other countries (Lemke et al., 2015; Lonely Planet (n.d.), 2020).

The country’s ostensibly tolerant and accepting attitudes toward sexual minorities may be a reflection of beliefs and practices dating back to before the arrival of Western settlers. Māori of pre-colonial New Zealand were said to celebrate sexual diversity, including same-gender relationships (Aspin, 2005). At a time when the puritanical views of the West saw homosexuality as something deviant, Māori had the concept of *takatāpui*, “*companion of the same sex,”* which was a normal part of early Māori culture. In contemporary New Zealand, takatāpui has come to represent the intersection of sexual and gender *fluidity*, and being Māori (Kerekere, 2015; Rainbow Youth, 2020).

In terms of adoption, New Zealand has a relatively simple adoption structure, in that virtually all adoption decisions—whether domestic or international—are facilitated and approved by the social workers in the former Adoption Unit, of the Ministry for Children (referred to locally by its Māori name: *Oranga Tamariki*). Unlike the United States, for example, New Zealand does not have both public and private adoption agencies; however, private adoptions can be undertaken with the aid of lawyers, but Oranga Tamariki will still have to approve the placements. Similarly, there are only a very small number of not-for-profit agencies (fewer than 10), accredited by Oranga Tamariki to act on their behalf in the matter of *international adoptions*—none of which are faith-based. The people who run these organizations do not work for the government, nor are they likely to be social workers; in many cases, they will be members of the adoption triangle, who became involved in order to help other New Zealanders become adoptive parents to overseas children. All adoptions facilitated by these accredited bodies, must still be vetted and approved by the social workers from Oranga Tamariki. New Zealand is also a signatory to the *Hague Convention on the Rights of the Child in Respect of Intercountry Adoption.*

New Zealand’s primary adoption legislation, which dates back to 1955, calls for ‘closed’ adoption practices. Despite this antiquated law, which is still in effect, Oranga Tamariki has long been facilitating open adoptions (Scherman, 2012). New Zealand is also a nation with a large population of people affected first-hand by adoption: in the late 1960s, New Zealand had one of the highest domestic adoption rates of the Western world, wherein more than 6% of its children were being placed for adoption (Iwanek, 1997).

Traditionally, Māori have had their own child placement practices, referred to as *whāngai*—a word meaning ‘to feed or nourish’ (Griffith, 1996; McRae and Nikora, 2006). For Māori, children are considered *taonga* (highly valued treasures); in this context, whāngai are essentially ‘gifted’ to the whāngai parent whose role it is to look after the children and nurture them through to adulthood (Else, 1991). Built on the importance of *whānau* and *whakapapa* (family and genealogy), this customary system has always been open, enabling the children to remain in contact with their birth parents (Walker, 2006; Gibbs and Scherman, 2013).

For those seeking to adopt, while a male individual cannot adopt a female child, there are no other barriers preventing LGBT persons from adopting children domestically. However, it was not until the passage of the *2013 Marriage Equality Act* that sexual minorities could adopt *as couples*. International adoptions, on the other hand, are not available to lesbian or gay individuals or couples: *“No countries we work with accept applications from couples in de facto, civil union or same-sex relationships; this results from the overseas countries’ legislation, policies and culture”* [ICANZ (Inter-Country Adoption New Zealand), 2020].

### Aim of the Current Study

The aim of our study was to examine lawyers’ and social workers’ perceptions of gay couples and lesbian couples adopting children. While legislation might make it possible for gay and lesbian couples to adopt, it remains unclear how these professionals perceive placing children with lesbian couples or gay couples. We focused on lawyers and social workers because they work in the fields that facilitate or assist adoptions and foster care placements. We reasoned that social workers would be the most obvious professionals to potentially engage with same-gender couples looking to foster or adopt children. However, there is a big gap in the literature on lawyers’ attitudes toward same-gender adoption. Because lawyers are often called on to facilitate private adoptions and are at the forefront of surrogacy arrangements, understanding their perceptions would be an important contribution to the literature. We guided our study with the following two research questions:

Research Question 1: What were New Zealand lawyers’ and social workers’ perceptions of same-gender adoption?

Research Question 2: How were the background characteristics of New Zealand lawyers and social workers related to their perceptions?

## Materials and Methods

### Participants

Upon receiving Institutional Review Board (IRB) approval from the first author’s university, participants were recruited via professional bodies and notices in professional newsletters. For social workers, we first approached their professional body, Aotearoa New Zealand Association of Social Workers, and asked them to pass along our invitation to participate. We also placed a small announcement in a monthly newsletter for social workers. In order to recruit lawyers, using the New Zealand Law Society website, we identified the practice areas with the greatest likelihood of potentially dealing with prospective adopters (e.g., family law), and then, with the aid of the law society, those lawyers were sent the study’s invitation and information sheet. The use of a third-party recruitment approach—as a mandate of the IRB—resulted in not knowing how many recruitment invitations were originally distributed. By extension, this meant that it was impossible to ascertain ‘response rates’ for either group of participants. In total, 313 online surveys were completed. Among them, 116 were lawyers and 173 were social workers. The other 24 respondents were in other fields such as psychotherapy and were removed from data analysis because of small sample size. There was no discernible difference between those with and without missing data in terms of available demographic information such as age, gender, occupation and ethnicity.

Table 1 summarizes the lawyers’ and the social workers’ background information. Both lawyers and social workers, on average, were middle-aged professionals who leaned toward the liberal end of the political spectrum. Overall, the two groups were not statistically different on five of the nine background variables (age, ethnicity, sexual orientation, relationship status, and parenting experience). There were significant or marginally significant differences on four variables: social workers scored significantly or marginally significantly higher on education level and political ideology than the lawyers; there were proportionately more females in the social workers; and there were marginally significantly more lawyers than social workers who reported identifying with some religion.

**TABLE 1:** Summary of demographic backgrounds between lawyers and social workers (*N* = 289).

Variable	Lawyers (*n* = 116)	Social workers (*n* = 173)	Chi-square			*t*-test		
			df	χ ^2^	*p*	df	*t*	*p*
Age	47.70 (*SD* = 10.50; *R* = 23–69)	49.50 (*SD* = 11.0; *R* = 24–71)				286	1.37	0.17
Education	5.47 (*SD* = 0.80; *R* = 5–7)	6.05 (*SD* = 0.95; *R* = 5–7)				287	5.43	0.00
Political ideology	4.80 (*SD* = 1.5; *R* = 2–7)	5.10 (*SD* = 1.2; *R* = 2–7)				287	1.96	0.06
**Ethnicity**			1	1.11	0.29			
European	105 (90.5%)	146 (86.4%)						
Non-European	11 (9.5%)	23 (13.6%)						
**Gender**			1	5.74	0.02			
Female	94 (81.0%)	157 (90.8%)						
Male	22 (19.0%)	16 (9.2%)						
**Sexual orientation**			1	2.53	0.11			
Heterosexual	108 (93.1%)	151 (87.3%)						
Non-heterosexual	8 (6.9%)	22 (12.7%)						
**Religion**			1	3.32	0.07			
No	62 (53.5%)	111 (64.2%)						
Yes	54 (46.7%)	62 (35.8%)						
**Relationship status**			1	1.61	0.20			
In a relationship	93 (80.2%)	126 (73.7%)						
Single	23 (19.8%)	45 (26.3%)						
**Parenting experience**			1	0.98	0.32			
Yes	87 (76.3%)	138 (81.2%)						
No	27 (23.7%)	32 (18.8%)						

### Procedures

We used an online survey hosted by Survey Monkey to gather data on the participants’ perceptions about placing children with same-gender couples. In all cases, invitations to participate directed interested persons to the Survey Monkey URL, wherein the first page of the survey explained that completion of the survey signaled consent. No one-on-one contact was made, nor was any identifying information collected, as dictated by the IRB. All participants completed the same survey.

### Instrument

#### Demographic Background

The participants responded to common demographic questions such as age, gender, ethnicity, educational background, occupation, and relationship status (married/in a long-term relation, single). Additionally, the participants responded to questions that were pertinent to the aims of the current study such as their sexual orientation (heterosexual, bisexual, gay, and lesbian) and whether the participant was a parent (yes and no). Finally, because existing literature has shown that both religiosity and political views are related to social attitudes toward homosexuality (Brown and Henriquez, 2008; Jäckle and Wenzelburger, 2015) and adoption (Perry, 2010), the participants responded to an open-ended question asking if they identified with any particular religion (*yes* or *no*) and another question about their political views (*1* = *extremely conservative, 2* = *moderately conservative, 3* = *slightly conservation, 4* = *neither conservative nor liberal, 5* = *slightly liberal, 6* = *moderately liberal, 7* = *extremely liberal*). In data analysis, these variables were treated as predictors of the participants’ perceptions of gay and lesbian adoption.

#### Perceptions of Adoption and Parenting by Gay Couples and Lesbian Couples

The participants were asked to respond to 24 statements on a 6-point Likert scale (*1* = *Strongly Disagree, 2* = *Moderately Disagree, 3* = *Slightly Disagree, 4* = *Slightly Agree, 5* = *Moderately Agree, 6* = *Strongly Agree*). This approach aimed to obtain information about the participants’ perceptions of gay and lesbian adoption (using heterosexual adoption as a “default” option), drawing inspiration for the statements from literature on lesbian women and gay men as sexual minorities, and current research focused on same-gender adoption and parenting. This section of the survey included both positive and negative statements. For example, *Same-gender relationships are as stable as heterosexual ones; If allowed to adopt, a lesbian or gay parent should only be allowed to adopt hard to place children*.

Post data collection inspection revealed that very few participants selected 1, 2, or 3 (strongly disagree, moderately disagree or slightly disagree). To reduce the imbalance, we truncated the response to be on a 3-point Likert scale by collapsing ratings of 1, 2, 3, and 4 into one category then rescaled the response to be on 0 (Strongly disagree, moderately disagree, slightly disagree, and slightly agree), 1 (moderately agree) and 2 (strongly agree). Exploratory factor analysis suggested that the items fell into three factors with high internal consistencies: *Equal Parenting Effectiveness* (12 items, α = 0.95), *Equal Opportunity to Adopt* (8 items, α = 0.81) and *Equal Treatment by Agency* (4 items, α = 0.80). The first factor (Equal Parenting Effectiveness) describes how much the participants believed that gay couples and lesbian couples were equally effective as heterosexual couples in parenting adopted children. The second factor (Equal Opportunity to Adopt) describes how much the participants believed that gay couples and lesbian couples should be given the same opportunity as heterosexual couples to adopt children. The third factor (Equal Treatment by Agency) describes how much the participants believed that agencies that place children for adoption should treat gay couples and lesbian couples equally as they treat heterosexual couples. The three factors are strongly correlated (*r*s = 0.66–0.78, *p* < 0.001). The scores could range from 0 to 3, with a higher score indicating a stronger endorsement. In data analysis, the mean of each factor was used.

#### Perception of Gay Couples’ and Lesbian Couples’ Suitability to Adopt Children With Special Needs

Because children waiting for adoption often have special needs and characteristics, we assessed how participants perceived the suitability of gay couples and lesbian couples to meet the challenges. In this assessment, we also included heterosexual couples for comparison. The participants indicated their extent of agreement (*1* = *Strongly Disagree, 2* = *Moderately Disagree, 3* = *Slightly Disagree, 4* = *Slightly Agree, 5* = *Moderately Agree, 6* = *Strongly Agree*) on seven hypothetical cases. The seven hypothetical cases were created for their typicality within the New Zealand care system, spanning low to high risk, with age and/or gender as added elements. They included (1) a child who needed to be placed with siblings, (2) a child who was a 13-year old teenager, (3) a healthy 8-year old girl, (4) a healthy 8-year old boy, (5) a sexually abused child who was sexually acting out, (6) a child who had chronic medical needs, and (7) a child with emotional and behavioral problems. For each child, the participants were asked to indicate their level of agreement of suitability for the child to be placed with a gay couple, a lesbian couple, and a heterosexual couple. The couples were described to be identical except their sexual orientation. For each type of couple, the participants’ responses to all the seven scenarios were averaged to reflect their perceptions. The internal consistency was 0.96 for the participants’ perceptions of the gay couple, 0.97 for the lesbian couple and 0.96 for the heterosexual couple. In data analysis, the mean for each type was used, the scores could range from 1 to 7 with higher scores indicated a stronger endorsement.

## Results

### Research Question 1: What Were New Zealand Lawyers’ and Social Workers’ Perceptions of Same-Gender Adoption?

As shown in Table 2, between-group comparisons using *t*-tests showed that the two groups did not score statistically differently on five of the six comparisons except that the lawyers scored lower than the social workers on whether gay couples and lesbian couples should have the same opportunity as heterosexual couples to adopt children.

**TABLE 2:** Summary of *t*-tests comparing means (SDs) of lawyers and social workers’ perceptions toward adoption by gay couples and lesbian couples and perception on their suitability to adopt children with special needs.

Variable	Lawyers	Social workers	df	*t*	*p*

General perception toward gay and lesbian adoption	*N* = 116	*N* = 173			
Equal parenting effectiveness as heterosexual couples	1.57 (0.56; *R* = 0–2)	1.58 (0.61; *R* = 0–2)	287	0.22	0.82
Equal opportunity to adopt as heterosexual couples	0.85 (0.51; *R* = 0–2)	1.02 (0.58; *R* = 0–2)	287	2.56	0.01
Equal treatment by agency as heterosexual couples	1.49 (0.59; *R* = 0–2)	1.58 (0.60; *R* = 0–2)	287	1.22	0.22

**Perception on suitability to adopt children with different needs**	***N* = 98**	***N* = 145**			

Gay couple	5.10 (1.33; *R* = 1–6)	5.32 (1.01; *R* = 1–6)	241	1.49	0.14
Lesbian couple	5.17 (1.21; *R* = 1–6)	5.36 (0.96; *R* = 1–6)	241	1.37	0.17
Heterosexual couple	5.68 (0.54; *R* = 1–6)	5.64 (0.53; *R* = 1–6)	241	0.56	0.57

Both lawyers and social workers scored relatively high on five of the six perceptions of gay and lesbian adoption, and on perceptions of whether gay couples, lesbian couples and heterosexual couples were suitable to adopted children with different needs. However, further within-group comparisons also showed significant differences. Specifically, in terms of general perceptions of gay and lesbian adoption, the lawyers scored an average of 1.57 (*SD* = 0.56) on whether they believed gay couples and lesbian couples were equally effective as heterosexual couples in raising adopted children, which is marginally higher than their scores of 1.49 (*SD* = 0.59) on whether they believed that adoption agencies should treat gay and lesbian couples equally as they treated heterosexual couples, *t*(*115*) = 1.86, *p* = 0.066. The lawyers’ average score of 0.85 (*SD* = 0.51) on whether gay and lesbian couples should have the same opportunity as heterosexual couples to adopt was significantly lower than their scores on whether the three types of couples were equally effective parents (*M* = 1.57, *SD* = 0.56), *t*(115) = 19.6, *p* < 0.001, and significantly lower than whether the three types of couples should be treated by the adoption agencies equally (*M* = 1.49, *SD* = 0.59), *t*(115) = 14.36, *p* < 0.001. Similarly, the social workers’ average score of 1.02 (*SD* = 0.58) that the three types of couples should have the same opportunity to adopt was significantly lower than their average scores on whether the three types of couples were equally effective in raising adopted children (*M* = 1.58; *SD* = 0.61) or whether they should be treated equally by adoption agencies (*M* = 1.58, *SD* = 0.60), *t*(172) = 15.27, *p* < 0.001.

In terms of beliefs about the suitability to adopt children with different needs, the lawyers scored an average of 5.68 (*SD* = 0.54) for heterosexual couples, which was significantly higher than their average score for gay couples (*M* = 5.10, *SD* = 1.33), *t*(97) = 4.02, *p* < 0.001, and for lesbian couples (*M* = 5.17, *SD* = 1.21), *t*(97) = 3.81, *p* < 0.001. However, they did not score differently on their perception of gay couples and lesbian couples (*M* = 5.10, *SD* = 1.33 versus *M* = 5.17, *SD* = 1.21), *t*(97) = 0.39, *p* = 0.70. For the social workers, the findings are similar: their average score for the suitability for heterosexual couples in adopting children with different needs was 5.64 (*SD* = 0.53), which was significantly higher than their average score for gay couples (*M* = 5.32, *SD* = 1.01), *t*(144) = 3.38, *p* < 0.001, and for lesbian couples (*M* = 5.17, *SD* = 1.21), *t*(144) = 3.07, *p* < 0.01. They did not perceive gay couples and lesbian couples differently (*M* = 5.32 versus 5.36), *t*(144) = 0.35, *p* = 0.73.

Overall, based on these results, New Zealand lawyers and social workers in our study reported generally favorable perceptions of adoption by gay couples and lesbian couples, but they favored heterosexual couples over gay couples and lesbian couples. Neither professional group made a distinction between their attitudes toward gay or lesbian couples.

### Research Question 2: How Were the Background Characteristics of New Zealand Lawyers and Social Workers Related to Their Perceptions?

To answer this question, we first obtained Pearson correlation coefficients between the participants’ background characteristics that were continuous (e.g., their age and political view) and their scores on their perceptions, then we reported the participants’ background characteristics that were categorical (e.g., male or female). The results are summarized in Tables 3–5.

**TABLE 3:** Correlations between participants’ background characteristics and their perceptions about adoption and special needs adoption by gay, lesbian and heterosexual couples (*N* = 243–289).

Variable	Lawyers			Social workers		
	Age	Education	Political ideology	Age	Education	Political ideology
**General perception toward gay and lesbian adoption**						
Equal parenting effectiveness as heterosexual couples	−0.20*	−0.25**	0.39***	−0.15*	0.08	0.18*
Equal opportunity to adopt as heterosexual couples	−0.25**	−0.16^∼^	0.41***	–0.12	0.06	0.21**
Equal treatment by agency as heterosexual couples	−0.18*	−0.17^∼^	0.33***	–0.11	0.03	0.12
**Perception on suitability to adopt children with different needs**						
Gay couple	–0.14	0.03	0.39***	−0.18*	–0.06	0.26**
Lesbian couple	–0.13	0.03	0.39***	−0.16^∼^	–0.05	0.27**
Heterosexual couple	–0.15	–0.03	0.14	–0.06	–0.03	0.08

^∼^p < 0.10, *p < 0.05, **p < 0.01, ***p < 0.001.

**TABLE 4:** Participants’ background characteristics and means (SD) of their general perceptions on adoption by gay and lesbian couples (*N* = 287).

	Equal parenting effectiveness		Equal opportunity to adopt		Equal treatment by agency	
Variable	Lawyers	Social workers	Lawyers	Social workers	Lawyers	Social workers
**Sex**						
Female	1.60 (0.53)	1.59 (0.61)	0.90 (0.52)	1.04 (0.57)	1.50 (0.59)	1.58 (0.60)
Male	1.41 (0.67)	1.45 (0.61)	0.64 (0.38)	0.81 (0.58)	1.44 (0.59)	1.53 (0.62)
df	114	171	114	171	114	171
*t*	1.46	0.91	2.20	1.50	0.40	0.31
*p*	0.15	0.37	0.03	0.14	0.69	0.75
**Ethnicity**						
European	1.55 (0.57)	1.60 (0.60)	0.83 (0.50)	1.04 (0.57)	1.48 (0.60)	1.61 (0.59)
Non-European	1.65 (0.41)	1.44 (0.73)	1.01 (0.56)	0.82 (0.57)	1.55 (0.52)	1.36 (0.69)
df	114	167	114	167	114	167
*t*	0.54	1.12	1.12	1.78	0.33	1.87
*p*	0.59	0.27	0.27	0.08	0.74	0.06
**Relationship status**						
Single	1.68 (0.38)	1.67 (0.51)	0.92 (0.53)	1.10 (0.58)	1.59 (0.57)	1.62 (0.53)
In relationship	1.53 (0.59)	1.56 (0.63)	0.83 (0.51)	0.99 (0.57)	1.47 (0.60)	1.58 (1.69)
df	114	169	114	169	114	169
*t*	1.16	1.12	0.80	1.10	0.88	0.34
*p*	0.25	0.26	0.43	0.27	0.39	0.73
**Sexual orientation**						
Heterosexual	1.56 (0.56)	1.53 (0.64)	0.84 (0.50)	0.98 (0.59)	1.47 (0.60)	1.53 (0.63)
Non-heterosexual	1.63 (0.48)	1.89 (0.16)	0.97 (0.62)	1.30 (0.39)	1.81 (0.44)	1.89 (0.18)
df	114	171	114	171	114	171
*t*	0.32	2.61	0.69	2.51	1.61	2.62
*p*	0.75	0.01	0.49	0.01	0.11	0.01
**Religiousness**						
No	1.66 (0.49)	1.73 (0.45)	0.90 (0.49)	1.12 (0.52)	1.54 (0.54)	1.64 (0.52)
Yes	1.45 (0.61)	1.32 (0.77)	0.79 (0.53)	0.82 (0.62)	1.43 (0.65)	1.46 (0.71)
df	114	171	114	171	114	171
*t*	2.09	4.37	1.16	3.46	1.08	1.92
*p*	0.04	0.00	0.25	0.00	0.28	0.06
**Parenting experience**						
No	1.65 (0.59)	1.73 (0.48)	1.07 (0.58)	1.11 (0.51)	1.59 (0.59)	1.68 (0.55)
Yes	1.53 (0.55)	1.55 (0.63)	0.78 (0.47)	1.00 (0.59)	1.47 (0.57)	1.57 (0.60)
df	112	168	112	168	112	168
*t*	1.02	1.51	2.67	1.01	0.97	0.97
*p*	0.31	0.13	0.01	0.31	0.33	0.33

**TABLE 5:** Participants’ background characteristics and means (SD) of their perceptions on the suitability for children with different characteristics to be adopted by gay, lesbian and heterosexual couples (*N* = 243).

Variable	Gay couple		Lesbian couple		Heterosexual couple	
	Lawyers	Social workers	Lawyers	Social workers	Lawyers	Social workers
**Sex**						
Female	5.21 (1.07)	5.36 (0.98)	5.28 (1.09)	5.40 (0.92)	5.66 (0.57)	5.64 (0.53)
Male	4.65 (1.52)	4.94 (1.29)	4.74 (1.54)	5.03 (1.27)	5.77 (0.39)	5.63 (0.51)
df	96	143	96	143	96	143
*t*	1.71	1.50	1.83	1.36	0.83	0.07
*p*	0.09	0.14	0.07	0.18	0.41	0.94
**Ethnicity**						
European	5.06 (1.35)	5.39 (0.94)	5.14 (1.24)	5.41 (0.93)	5.68 (0.55)	5.65 (0.51)
Non-European	5.37 (1.09)	4.80 (1.35)	5.41 (0.91)	4.99 (1.18)	5.71 (0.46)	5.54 (0.63)
df	96	139	96	139	96	139
*t*	0.73	2.41	0.71	1.74	0.22	0.86
*p*	0.46	0.02	0.48	0.08	0.83	0.39
**Relationship status**						
Single	5.16 (1.14)	5.34 (0.94)	5.27 (0.98)	5.37 (0.94)	5.59 (0.66)	5.59 (0.56)
In Relationship	5.08 (1.38)	5.31 (1.04)	5.14 (1.26)	5.35 (0.98)	5.70 (0.51)	5.66 (0.51)
df	96	142	96	142	96	142
*t*	0.22	0.15	0.43	0.09	0.84	0.68
*p*	0.82	0.88	0.67	0.93	0.40	0.50
**Sexual orientation**						
Heterosexual	5.09 (1.35)	5.25 (1.06)	5.17 (1.22)	5.30 (1.00)	5.70 (0.51)	5.62 (0.53)
Non-heterosexual	5.18 (1.18)	5.73 (0.54)	5.23 (0.99)	5.71 (0.54)	5.43 (0.85)	5.74 (0.53)
df	96	143	96	143	96	143
*t*	0.18	2.00	0.15	1.80	1.37	0.94
*p*	0.86	0.04	0.88	0.07	0.17	0.35
**Religiousness**						
No	5.34 (1.14)	5.49 (0.72)	5.36 (1.06)	5.51 (0.67)	5.73 (0.53)	5.65 (0.53)
Yes	4.83 (1.47)	4.98 (1.40)	4.96 (1.32)	5.05 (1.34)	5.63 (0.56)	5.62 (0.52)
df	96	143	96	143	96	143
*t*	1.96	3.82	1.67	2.78	0.95	0.29
*p*	0.05	0.00	0.10	0.01	0.34	0.77
**Parenting experience**						
No	5.49 (0.94)	5.66 (0.56)	5.51 (0.83)	5.63 (0.61)	5.66 (0.59)	5.78 (0.40)
Yes	4.98 (1.42)	5.24 (1.08)	5.06 (1.29)	5.29 (1.02)	5.69 (0.54)	5.60 (0.54)
df	94	141	94	141	94	141
*t*	1.58	1.95	1.54	1.66	0.17	1.55
*p*	0.12	0.05	0.13	0.10	0.86	0.12

As shown in Table 3, there were both similarities and differences. For both lawyers and social workers, more liberal political ideology was significantly and positively correlated with higher scores on most of the six perceptions. However, their educational level was mostly uncorrelated with their perceptions. Among the lawyers, being older was correlated with lesser belief in same-gender couples’ parenting effectiveness; lesser belief in the same-gender couples getting equal opportunities to adopt; and lesser belief that the same-gender couples should get equal treatment by agencies.

As shown in Tables 4, 5, most of the background characteristics were not significant for either lawyers or social workers. However, several background variables such as the participants’ sexual orientation and religiousness were related to the participants’ scores on their perceptions (for details, see Tables 4, 5).

To determine how the background characteristics jointly affected the lawyers’ and social workers’ perceptions, we subsequently ran multiple regression analyses for scores of each of the six outcome measures using two regression models. In the initial model, all background variables were entered into the regression at once, in the final model, only significant predictors were retained to identify a parsimonious set of significant predictors. We additionally tested interactions but none were significant. The results are summarized in Tables 6, 7. When these variables were entered into the initial regression models simultaneously, more liberal political ideology and a lack of religion predicted more favorable perceptions. The participants’ occupation, age and ethnicity were not significant in predicting any of the five outcome variables. Other variables such as gender, sexual orientation, relationship status, and parenting experiences were significant in predicting some aspects of the participants’ perceptions. In the final models, political ideology, sexual orientation and religiousness were the most consistent predictors of the participants’ scores on whether they believed that gay couples, lesbian couples, and heterosexual couples were equally effective as adoptive parents, whether the three types of couples should have the opportunity to adopt and whether they should be treated equally by adoption agencies. Interestingly, political ideology and religiousness were significant in predicting the participants’ scores on whether they believed that gay couples and lesbian couples were suitable to adopt children with different special needs, but none of the variables predicted the participants’ scores on whether they believed that heterosexual couples were suitable to adopt children with different special needs (for details see Tables 6, 7).

**TABLE 6:** Summary of regression analysis predicting lawyers and social workers’ general perceptions on adoption by lesbian couples and gay couples (*N* = 289).

	Equal parenting effectiveness		Equal opportunity to adopt		Equal treatment by agency	
	Initial model	Final model	Initial model	Final model	Initial model	Final model
Intercept	1.63***	1.51***	0.54	0.34*	1.63***	1.37***
Age	0		0		–0.01	
Education level	−0.08*	−0.08*	0		–0.03	
Political ideology	0.09***	0.10***	0.09***	0.10***	0.07**	0.09***
**Sex**						
Female	0.11		0.17*	0.23**	0.03	
Male	Referent (0)		Referent (0)	Referent (0)	Referent (0)	
**Ethnicity**						
European	0.03		0.05		0.13	
Non-European	Referent (0)		Referent (0)		Referent (0)	
**Relationship status**						
Single	0.17*	0.14*	0.11		0.10	
In relationship	Referent (0)	Referent (0)	Referent (0)		Referent (0)	
**Sexual orientation**						
Heterosexual	−0.16*	−0.16*	−0.16^∼^	−0.22*	−0.26***	−0.31***
Non-heterosexual	Referent (0)	Referent (0)	Referent (0)	Referent (0)	Referent (0)	Referent (0)
**Religiousness**						
No	0.23**	0.24***	0.14*	0.17**	0.04	
Yes	Referent (0)	Referent (0)	Referent (0)	Referent (0)	Referent (0)	
**Parenting experience**						
No	0.02		–0.09		0	
Yes	Referent (0)		Referent (0)		Referent (0)	
**Profession**						
Lawyers	–0.01		−0.11^∼^		–0.08	
Social workers	Referent (0)		Referent (0)		Referent (0)	
*df*	(10, 277)	(5, 286)	(10, 277)	(4, 288)	(10, 277)	(2, 286)
***F***	4.59***	8.52***	7.18***	16.51***	5.13***	23.90***
***R*^2^**	0.17	0.15	0.17	0.15	0.10	0.07

^∼^p < 0.10, *p < 0.05, **p < 0.01, ***p < 0.001.

**TABLE 7:** Summary of regression analysis predicting lawyers and social workers’ perceptions on the suitability of lesbian couples, gay couples and heterosexual couples in adopting children with different characteristics (*N* = 243).

	Gay couples		Lesbian couples		Heterosexual couples	
	Initial model	Final model	Initial model	Final model	Initial model	Final model
Intercept	3.88***	3.66***	4.02***	3.82***	5.78***	5.46***
Age	–0.01		0		0	
Education level	–0.01		–0.01		–0.02	
Political ideology	0.23***	0.25***	0.22***	0.23***	0.04	0.04^∼^
**Sex**						
Female	0.40		0.37		–0.12	
Male	Referent (0)		Referent (0)		Referent (0)	
**Ethnicity**						
European	0.13		0.02		0.03	
Non-European	Referent (0)		Referent (0)		Referent (0)	
**Relationship status**						
Single	0.05		0.05		–0.06	
In relationship	Referent (0)		Referent (0)		Referent (0)	
**Sexual orientation**						
Heterosexual	–0.19		–0.16		0.02	
Non-heterosexual	Referent (0)		Referent (0)		Referent (0)	
**Religiousness**						
No	0.39*	0.37*	0.34*	0.31*	0	
Yes	Referent (0)	Referent (0)	Referent (0)	Referent (0)	Referent (0)	
**Parenting experience**						
No	0.23	0.32*	0.18	0.26*	0.05	
Yes	Referent (0)	Referent (0)	Referent (0)	Referent (0)	Referent (0)	
**Profession**						
Lawyers	–0.04		–0.02		0.03	
Social workers	Referent (0)		Referent (0)		Referent (0)	
*df*	(10, 233)	(3, 228)	(10, 233)	(3, 228)	(10, 233)	(1, 242)
*F*	4.05***	1.81***	3.40***	9.16***	0.76	2.70
*R*^2^	0.18	0.15	0.17	0.15	0.03	0.01

Overall, our regression analyses suggest that a stronger liberal political ideology and a lack of religiousness were the two most consistent predictors of the participants’ positive perceptions of lesbian and gay adoption.

## Discussion

The aim of this study was to examine the perceptions of lawyers and social workers about children being placed with same-gender adoptive parents. In choosing to carry out the study in New Zealand, we wanted to take advantage of relatively recent statutory changes that legalized same-gender marriage— which by extension, made it possible for same-gender couples to adopt. Additionally, with New Zealand being widely perceived as a progressive and inclusive country with regard to its LGBT communities, the location afforded us a unique opportunity to passively consider possible environmental influences. In short, carrying out the research here made us hopeful that we would see the country’s egalitarian ideals mirrored in the professionals’ perceptions. The findings paint a mixed picture that requires some teasing out.

### The Persistent Influence of Religiosity and Political Ideology

When looking broadly across the demographic variables, we found a similar pattern of results to the international literature: being religious and having conservative political leanings were characteristics associated with more negative perceptions toward placing children with same-gender couples. These findings lend support to the many studies that have identified religiosity as a key feature of more bias beliefs about lesbian and gay adopters (Brodzinsky, 2003; Jayaratne et al., 2008; Mallinger, 2010; McCutcheon and Morrison, 2014; Jäckle and Wenzelburger, 2015; Kimberly and Moore, 2015). Our findings on the relationship between biases against same-gender adoptions and the holding of conservative ideologies fit also with the many studies that have found political views to be related to social attitudes toward homosexuality (Brown and Henriquez, 2008; Jäckle and Wenzelburger, 2015) and adoption (Hall, 2010; Perry, 2010)—and studies that have found both attributes present in persons with more negative perceptions of adoptions by couples of the same-gender (Costa et al., 2014; Molina and Alarcón, 2015; Takács et al., 2016).

### Movement Toward More Positive Views, or Hidden Biases?

With regard to the exploratory factor analysis, and the three factors described in the Findings section, our data also mirrors much of the international research, but with some subtle caveats. As noted earlier, the main questionnaire fell into three factors: Parenting Effectiveness (belief that homosexual and heterosexual parents could be equally effective), Opportunity to Adopt (belief that gay and lesbian individuals should be given the same opportunities as heterosexual individuals to adopt), and Placement Agency Treatment (belief that agencies should treat heterosexual and homosexual applicants equally). The findings show that collectively both lawyers and social workers felt strongly that gay and lesbian parenting is as effective as heterosexual parenting, and that placement agencies should treat prospective adopters in same-gender relationships as they would treat prospective adopters who are in heterosexual relationships—both positive and promising outcomes. However, in terms of the third factor, participants did not strongly endorse the idea that lesbian and gay couples should be given the same opportunities as heterosexual couples to adopt children. Why would the participants agree that same-gender couples parent on par with heterosexual couples, and that the lesbian and gay couples should not be treated differently to heterosexual couples, but then say that the same-gender couples should not be given the same opportunities to adopt?

We found this outcome surprising, and speculated that the two affirmative factors could be described as more concrete ideas, enshrined as they are in research evidence (in terms of parenting abilities) and legislative mandates (requiring that sexual minorities not be discriminated against), which may represent more objective, modern thinking. On the other hand, believing that lesbian and gay individuals should be given the same *opportunities* as heterosexual individuals—or not, in the case of this study, may reflect more subjective beliefs, that are less concrete and more emotive in nature.

We wondered further if the incongruity of these three factors could be an example of *modern* prejudices against lesbian and gay couples wishing to adopt. This social psychological concept, originally identified in the context of racism (McConahay et al., 1981), is subtler and more covert, quite unlike old fashioned or traditional prejudices that were blatant, pejorative, and hostile. However, with modern prejudice, people frequently believe that they are not prejudice, even expressing more egalitarian views (something we saw with the first two factors), which suggests that modern prejudice may reflect unconscious attitudes. This idea aligns somewhat with what Sullivan and Harrington (2009) described as *vicarious stigma*, when the social workers acted in the interests of biases they expected to exist.

Modern prejudice has also been theorized as an unintentional unwillingness to help. Banaji and Greenwald (2016) explained that this “not helping” can come in the form of in-group favoritism, but without necessarily realizing it; and since there is no overt prejudices, it can look innocent enough. Yet, this standard of not helping, then strengthens existing patterns of disadvantage (Banaji and Greenwald, 2016). Applying these theoretical ideas to the current study, the third less-endorsed factor measuring ideas about equal treatment by agency, could be perceived as a type of helping response; and according to modern prejudice, this not wanting to help can sit comfortably alongside the positive perceptions of the first two factors. Ultimately, however, we did not explicitly explore possible modern prejudices within the attitudes toward adoption by same-gender, so any further consideration will require additional research^1^.

### A (Rare) Look at the Perceptions of Lawyers

As discussed in the review of research, lawyers are among the professionals that likely work with sexual minorities in their bid to become parents. For that reason, their perceptions are also important to explore, hence their inclusion in the current study. And although they have much to say about the topic of adoption by same-gender couples, and have published extensively on the subject, it still came as a surprise to find no attitudinal research with lawyers. The surprise was short-lived, however, as we quickly concluded (with a bit of a chagrin) that lawyers would be interested in the laws surrounding same-gender adoption; and while the law articles greatly concerned themselves with (e.g.) the “best interests of the children” and other human interests, as a profession, we suspect that lawyers are not as interested in the thoughts and feelings one might have toward adoptions by same-gender couples. It is for this reason that we were especially pleased to get such a good survey response (*n* = 116) from the lawyers.

When considered as a group, and compared to the social workers, the data showed that lawyers did not differ on any of the outcome measures. The one exception to this is that lawyers scored lower than social workers on their beliefs that lesbian couples and gay couples should have the same opportunities as heterosexual couples to adopt when none of the covariates were considered (Table 4). When the covariates (age, education, political ideology, gender, and ethnicity) were considered (Table 6), the difference was reduced to a non- significant trend. In light of the above-consideration of this overall finding—that it might reflect the differences between objective and subjective beliefs. We cautiously speculate that legal training might influence lawyers to prioritize objective ideas over subjective ones, whereas social workers might instead prioritize feelings and subjectivity leading to subtle differences in perspective between the two professional groups.

In terms of the secondary speculation, that this lower score in the third factor might reflect modern prejudice, we have no reason to believe that lawyers would be any more (or less) prejudiced than social workers. However, when one considers the law literature on same-gender parenting, and the strong emphasis on ensuring the best interests of the children, lawyers might be more inclined to argue from the children’s perspectives. This perspective might predispose the lawyers to agree with some of the myths about children being harmed or disadvantaged, especially as there is research evidence that children being raised by same-gender parents do sometimes experience poorer outcomes (Crouch et al., 2016; Cenegy et al., 2018), but due to largely demographic and socioeconomic differences rather than exposure to a non-traditional family form (Misca and Smith, 2014). Lastly, it is the lawyers who are the most aware of the legal ramifications and complexities of same-gender adoption, including the risks associated with having a silent, non-legal parent, all of which could influence their beliefs about treating homosexual couples the same as heterosexual couples. Having no previous attitudinal literature to draw upon with regard to the perceptions of lawyers has necessarily limited our consideration of our findings. We argue strongly that much more research is needed on the attitudes, beliefs and perceptions of lawyers and others in the legal and criminal justice sectors, as pertains same-gender parenting.

As a final discussion point, we reflect on the consistency in the data from the hypothetical cases, in terms of the finding that regardless of the children’s backgrounds and characteristics, both sets of participants collectively preferred to see children placed with heterosexual couples over same-gender couples. Neither New Zealand lawyers nor social workers appeared to distinguish between lesbian and gay couples applying to adopt children. Finding that the participants favored heterosexual couples over homosexual couples reflects the well-established hierarchy of preference seen in the literature, which places heterosexual couples first (Stacey, 2006). However, finding that the two groups did not differ in their preference between gay couples and lesbian couples, was incongruent with the literature, since, as part of that hierarchy of preference, lesbian couples are usually favored over gay couples (e.g., Gianino, 2008; Tuazon-McCheyne, 2010). Their violation of traditional gender roles, is thought to be why gay men as parents receive more criticism and suspicion, compared to lesbian women as parents (Carneiro et al., 2017). Thus, our finding that the New Zealand lawyers and social workers did not perceive gay couples and lesbian couples differently in their ability to adopt children of different needs, confirms that the New Zealand context might be unique in comparison to other places. We speculate that the proportionately fewer participants who follow a religion, and possible shifts in the perceptions and acceptance of gay couples in New Zealand might have played a role. More research is needed to confirm these speculations.

### Strengths, Limitations and Future Research

The findings of our study need to be cautiously interpreted within its limitations. As a cross-sectional survey study, it has the typical limitation of relying on volunteers, which prevents us from generalizing findings to other professionals in New Zealand or in other countries. Moreover, the study was set up as an exploratory inquiry into the perceptions of a range of professionals involved in the adoption process. The exploratory stance allowed us to tap into a category of professionals not previously explored in this context—lawyers, and to examine their perceptions of same-gender adoption, as compared to a cohort of social workers. As reported here, both groups held similar perceptions of adoption by same-gender and opposite-gender couples; yet subtle differences were reflected in the lawyers’ stronger perceptions that same-gender couples should not be given the same opportunities to adopt as heterosexual couples. Findings like this warrant further investigation given the crucial role lawyers play in not only the adoption process, but also in other related domains like surrogacy—an alternative method of family formation also sought out by lesbian and gay couples wishing to start families.

On the other hand, the broad stance of our design did not allow for in-depth delving into what might be specific to this group of lawyers or what may influence their perceptions of same-gender adoptions. Thus, we believe that much more research with lawyers is needed, some of which takes an in-depth look at, for example, their perceptions, attitudes, and assumptions, leading to better understanding of the subtle differences seen between them and social workers in the current study.

Future research should also look to include more diverse groups of professionals who work with prospective adopters, such as clinicians, educators, or health professionals. Moreover, future studies would benefit from expanding into different cultural and legal contexts, where broader sociocultural and statutory influences might be more centrally explored. The present study took place in a country known to be more tolerant of lesbian and gay communities, but without explicitly testing those sampled for their degree of tolerance *per se*. Finally, continued research in this area of study would benefit from developing theoretical models of how implicit and explicit attitudes toward sexual identity and parenting develop; and how they then “overspill” into professional practice (Tan et al., 2017). Understanding such pathways, through which personal perceptions, beliefs and attitudes influence professionals, are crucial for improving training and professional practice, yet this understanding is still missing.

A notable contribution of our study is the development of an instrument designed to capture the beliefs of professionals in regards to same-gender prospective adopters and its initial validation analyses reported in this paper indicates good potential. The authors are interested in further validating the instrument in different countries/cultures and with different groups of professionals, and invite potential interested researchers to contact them (At the time of writing there are plans underway for the study to be replicated in Quebec, Canada and thus the instrument to be translated into French).

### Implications

Adoption as a legal phenomenon, creates new parenthood but importantly, the children’s needs drive the processes and determine the adoptive parents’ suitability; thus prospective adoptive parents are being selected to meet the needs of a specific child (Scherman et al., 2016). This may suggest the need for training for lawyers, beyond following the letter of the law, but which also includes an understanding of ways in which different types of family can effectively support children. The law literature emphatically highlights the potential risks to the parent/child relationship but the law articles do so from a statutory perspective. Understanding of the parental relationship from a socioemotional perspective would provide valuable insights and lead to improved practice to ensure the best interest of the child (Zarembka, 2015).

Furthermore, lawyers and social workers may potentially benefit from shared or integrated training and practice. In social work curriculum worldwide, law is a universal presence and important component of training social workers (Sewpaul and Jones, 2005); yet in law training, it is rarely the case that social science curriculum is incorporated, although sorely needed (AFCC Task Force on the Guidelines for the Use of Social Science in Family Law, 2019).

## Conclusion

Returning to the question raised at the beginning of the paper, of whether the statutory changes allowing same-gender marriage will be enough on their own to shift the social norms about same-gender adoption, the answer would appear to be no— even though the former is required in New Zealand, as in some other countries, for the latter to occur. Winning the rights to marry appears to be just the first hurdle that must be overcome; normalizing same-sex parenting is what needs to happen next.

Taken together, our findings underscore the value of examining multiple perceptions about same-gender parenting in the adoption context; and point out that even in the context of a country with seemingly progressive attitudes and policies toward sexual minorities, there is still progress to be made in mitigating discrimination against same-gender couples seeking to adopt. Our study adds to the evidence from studies of professionals in other countries such as those in Spain, the United States, and Canada, with the ultimate aim to inform practices and policies that support lesbian and gay couples seeking to form families through adoption.

## Data Availability Statement

The datasets generated for this study are available on request to the corresponding author.

## Ethics Statement

The studies involving human participants were reviewed and approved by Auckland University of Technology Ethics Committee (approval number 14/404). Written informed consent for participation was not required for this study in accordance with the national legislation and the institutional requirements.

## Author Contributions

RS and GM developed the study and the instrument/survey. RS led the data collection, the literature review, and the writing of the manuscript. TXT analyzed the data. All authors contributed to and approved the final manuscript.

## Conflict of Interest

The authors declare that the research was conducted in the absence of any commercial or financial relationships that could be construed as a potential conflict of interest.

## Footnotes

1 Incidentally, several researchers have already begun to explore modern prejudice toward lesbian women and gay men, and same-sex parenting (e.g., Morrison and Morrison, 2003; Massey et al., 2013).
